# Diversity of *Weissella confusa* in *Pozol* and Its Carbohydrate Metabolism

**DOI:** 10.3389/fmicb.2021.629449

**Published:** 2021-03-18

**Authors:** Diana Hernández-Oaxaca, Rafael López-Sánchez, Luis Lozano, Carmen Wacher-Rodarte, Lorenzo Segovia, Agustín López Munguía

**Affiliations:** ^1^Departamento de Ingeniería Celular y Biocatálisis, Instituto de Biotecnología, Universidad Nacional Autónoma de México (UNAM), Cuernavaca, Mexico; ^2^Programa de Genómica Evolutiva, Centro de Ciencias Genómicas, Universidad Nacional Autónoma de México (UNAM), Cuernavaca, Mexico; ^3^Departamento de Alimentos y Biotecnología, Facultad de Química, Universidad Nacional Autónoma de México (UNAM), Ciudad de México, Mexico

**Keywords:** *pozol*, maize, *Weissella*, fermented foods, CAZy

## Abstract

The genus *Weissella* is composed of a group of Gram-positive facultative anaerobe bacteria with fermentative metabolism. Strains of this genus have been isolated from various ecological niches, including a wide variety of fermented cereal foods. The present study aimed to determine the relative abundance and fermentation capabilities of *Weissella* species isolated from *pozol*, a traditional *maya* product made of lime-cooked (nixtamalized) fermented maize. We sequenced the V3-V4 regions of 16S rDNA; *Weissella* was detected early in the fermentation process and reached its highest relative abundance (3.89%) after 3 h of culture. In addition, we evaluated five *Weissella* strains previously isolated from *pozol* but reported as non-amylolytic, to define alternative carbon sources such as xylan, xylooligosaccharides, and sucrose. While no growth was observed on birch xylan, growth did occur on xylooligosaccharides and sucrose. Strains WcL17 and WCP-3A were selected for genomic sequencing, as the former shows efficient growth on xylooligosaccharides and the latter displays high glycosyltransferase (GTF) activity. Genomes of both strains were assembled and recorded, with a total of 2.3 Mb in 30 contigs for WcL17 and 2.2 Mb in 45 contigs for WCP-3a. Both strains were taxonomically assigned to *Weissella confusa* and genomic analyses were performed to evaluate the gene products encoding active carbohydrate enzymes (CAZy). Both strains have the gene content needed to metabolize sucrose, hemicellulose, cellulose, and starch residues, all available in *pozol*. Our results suggest that the range of secondary enzymatic activity in *Weissella confusa* strains confer them with wide capabilities to participate in fermentative processes of natural products with heterogeneous carbon sources.

## Introduction

*Pozol*, from the Nahuatl *pozolli*, is an acidic beverage obtained from the non-alcoholic fermentation of *nixtamalized* (lime-cooked) maize. In ancient times, it was one of the most important cultural components used by peninsular Mayans in offerings to the gods and spirits in ceremonies related to maize cultivation and harvest (Vassallo Rodríguez, 2016). It is currently consumed in southeastern Mexico, where it remains as a food of nutritional, cultural, and economic importance (Sangwan et al., 2014).

The microbial complexity involved in *pozol* fermentation has been studied for several decades using microbiological, biochemical, and molecular techniques, as there is no established inoculation procedure. A number of facts are currently known in this regard. On the one hand, lactic acid bacteria (LAB) are dominant in all stages of *pozol* fermentation (Wacher et al., 1993; Nuraida et al., 1995; ben Omar and Ampe, 2000). On the other, the microbiota involved in *pozol* fermentation is complex, including filamentous fungi and yeasts observed in outer layers, while bacteria are found mainly at the core of the dough ball (Ampe et al., 1999a). Multiple bacterial genera have been identified in *pozol*, with *Streptococcus* and *Weissella* being frequently mentioned in the scientific literature on *pozol* (Ampe et al., 1999b; ben Omar and Ampe, 2000; Wacher et al., 2000; Escalante et al., 2001; Bolaños et al., 2006; Cárdenas et al., 2014). Strains isolated from *pozol* synthesize broad-spectrum antimicrobial compounds that can be used as food preservatives (Ray et al., 2000; Phister et al., 2004). Moreover, exopolysaccharides synthesized from sucrose may function as soluble fiber or prebiotics (Olivares-Illana et al., 2002).

As regards the source of carbon, starch is the main carbohydrate available in nixtamalized maize. In this sense, amylolytic lactic-acid bacteria (ALAB) in the genera *Enterococcus, Lactococcus* and *Streptococcus* have been identified in *pozol*, with *S. bovis* (*Streptococcus infantarius* ssp. *infantarius*) as the species with the highest specific growth rate on starch, but with low amylase activity relative to other ALAB species (Díaz-Ruiz et al., 2003). No amylase activity was found in *Weissella* strains (López-Hernández et al., 2018), as also reported for *Weissella* strains in other cereal-based fermented foods (Sharma et al., 2018).

It has been suggested that the hemicellulose available after nixtamalization may be an alternative substrate for dough fermentation. In this context, it was recently demonstrated that *S. infantarius* isolated from *pozol* can assimilate xylan in *nejayote* (residual water from nixtamalization) and xylan from birch wood through xylanolytic activity (Cooper-Bribiesca et al., 2018). Similarly, *W. confusa* L9 strain assimilates xylooligosaccharides (XOS) in nixtamalized corn (López-Hernández et al., 2018). Activities of this sort have been identified in sourdough fermentation, for which the destructuring of insoluble fiber and an increase in soluble dietary fiber after fermentation have been reported (Decimo et al., 2017). Furthermore, the consumption of XOS by strains of this genus isolated from Indian fermented foods has also been described (Patel et al., 2013).

Interestingly, *Weissella* strains have been isolated from highly diverse fermented products (Fusco et al., 2015), including jeotgal and kimchi, nono and douchi, among many others (Lee et al., 2010; Ayeni et al., 2011; Liu et al., 2012). Moreover, the presence of *W. confusa* in multiple types of fermented food with cereal as fermentation matrix is a remarkable finding (Ampe et al., 1999b; Mugula et al., 2003; Abegaz, 2007; Bounaix et al., 2010; Oguntoyinbo et al., 2011; Owusu-Kwarteng et al., 2012; Elizaquível et al., 2015; Osimani et al., 2015; Kavitake et al., 2016). Furthermore, several *Weissella* strains isolated from fermented foods produce exopolysaccharides from sucrose, particularly dextran (Tieking et al., 2003; Park et al., 2013; Malang et al., 2015; Shukla et al., 2016; Rizzello et al., 2019). Dextran has also been detected in products made from wheat and rye bran fermented by two *Weissella confusa* strains isolated from fermented vegetables (Kajala et al., 2016), and in quinoa-flour yogurt added with sucrose (Lorusso et al., 2018). This ability to synthesize exopolysaccharides with soluble-fiber or prebiotic potential through glycosyltransferase (GTF) activity is an additional interesting feature of this genus (Malang et al., 2015).

Based on this background, we evaluated the importance of the genus *Weissella* in terms of its presence throughout the *pozol* fermentation process. In addition, we explored the biochemical and genomic ability of *Weissella* strains isolated from *pozol* to assimilate birch xylan, XOS, and sucrose as alternative carbon sources in *pozol* fermentation, as well as the presence of GTF activity.

## Materials and Methods

### Mass Sequencing of the 16S rDNA Marker in *Pozol* Samples

*Pozol* samples were prepared as described in Rizo et al. (2020). Freshly ground nixtamal dough samples were obtained from two producers at the Pino Suárez market in Tabasco, Mexico. Triplicate samples were mixed and shaped into 300-g balls, wrapped in banana leaves, and incubated at 37°C. Sampling was performed at 0, 9, 24, and 48 h, and handled aseptically. DNA was extracted with commercial kits (PowerSoil DNA Isolation Kit, PowerMax Soil DNA Isolation Kit, and UltraClean Microbial DNA Isolation Kit (MO BIO, QIAGEN). DNA was purified and, using specific oligonucleotides (Klindworth et al., 2013), the V3-V4 region of the 16S rDNA marker was amplified with Phusion High-Fidelity DNA Polymerase (Thermo Scientific). Amplicons were sequenced on an Illumina MiSeq (2 × 300 bp) platform. Amplicons were reconstructed with Flash v1.2.7 (Magoč and Salzberg, 2011). All non-overlapping sequences were excluded from further analyses. The extended fragments were dereplicated, chimeras were removed, and singletons were filtered with VSEARCH-v2.4.3 (Rognes et al., 2016), generating an OTUs matrix of 97% similarity. The taxonomic annotation was conducted with the ParallelMeta v2.4.1 software (Su et al., 2014) and the Metaxa2 v2.1.1 software (Bengtsson-Palme et al., 2015), as described in Escobar-Zepeda et al. (2018). Rarefaction curves and alpha diversity indexes were prepared with the R libraries metagenomeSeq (Paulson et al., 2013) and phyloseq (McMurdie and Holmes, 2013). An NDMS plot was constructed using the dissimilitude Bray-Curtis index with the vegan package in R (Oksanen et al., 2012).

### Microorganisms and Culture Conditions

Five *Weissella* strains (WcSnc45, WcSnc40, WcL9, WcL17, and WCP-3a) isolated from *pozol* were included in this study López-Hernández et al. (2018). These strains were preserved in glycerol at −80°C. A 10% (v/v) aliquot was inoculated to De Man Rogosa and Sharpe (MRS) medium (Diffco, United States), which was incubated at 30°C for 24 h. Afterward, a 10% (v/v) aliquot was transferred to MRS broth and incubated at 30°C for 12 h; the latter was used as inoculum for fermentation experiments. Additionally, a 0.6% (v/v) aliquot was inoculated in 1% modified glucose-free MRS broth, prepared by adding three carbon sources: birch xylan, xylooligosaccharides (XOS), and sucrose, as proposed by López-Hernández et al. (2018). The media were incubated at 30°C for 48 h; samples were collected at different times over the culture period to observe the evolution of pH and measure optical density (OD) at 600 nm, used to calculate the growth rate. The culture supernatant was recovered by centrifugation at 16 000 g for 10 min and then transferred to two Eppendorf tubes, one of which was inactivated by heating in boiling water for 10 min; both tubes were stored at −4°C.

### Growth Kinetics in Birch Xylan, Xylooligosaccharides, and Sucrose

Growth of the five strains in MRS-birch xylan, MRS-XOS, and MRS-sucrose was followed for 48 h, with the non-inactivated enzymatic extract used to measure enzymatic activity. Biological and experimental replicates were run in duplicate in all tests and analyzed using the ggpubr package in R v4.0.2. Significant differences were examined with a Tukey *post hoc* test after a Kruskal-Wallis test. The detailed procedure is described in the repository, Data Availability section. Xylanolytic and GTF activities were determined through the quantification of reducing sugars using the dinitrosalicylic acid (DNS) method (Miller, 1959). A standard xylose curve (Sigma, United States) (0–2 g/L) was used as reference for xylanolytic activity calculations. The reaction mixture contained 300 μL of substrate (0.5% birch xylan in 0.1 M acetate buffer, pH 5.3) and 300 μL of culture supernatant. The reaction was incubated at 50°C and 800 rpm for 6 h, collecting 50 μL reaction samples at 0, 3, and 6 h. A standard curve of glucose and fructose (Sigma, United States) (0–2 g/L) was used as reference for GTF activity measurements. The reaction mixture contained 50 μL of 60% sucrose, 150 μL of 50 mM acetate buffer, pH 6.0, and 300 μL of supernatant. The reaction was incubated at 37°C and stopped at 0, 10, 20, and 30 min. In both cases, the enzyme was inactivated by adding 50 μL of DNS and then used to measure the reducing sugars. The sample was incubated at boiling temperature for 5 min and transferred to ice for 5 min. Five-hundred milliliters of distilled water were added, and absorbance was read at 540 nm using the 0 h time point as blank.

As regards the kinetics with MRS-sucrose, the strains that showed the greatest growth were re-evaluated. The inactivated supernatants were used to determine the amount of sucrose metabolized throughout the fermentation process by high-performance liquid chromatography (HPLC) and polymer formation by gel permeation chromatography (GPC), as reported by Porras-Domínguez et al. (2015). Xyloligosaccharides were produced from birch xylan following the procedure of Akpinar et al. (2009) and later used as substrate to measure xylosidase activity and for kinetics studies in MRS-XOS *Weissella* cultures. Based on these results, the strain showing the highest growth (WcL17) was selected for further studies. Samples of the inactive culture extract were used to evaluate the metabolized XOS profile by thin-layer chromatography (TLC) in silica gel-coated aluminum plates (Merck, Germany). To this end, 20 μg of sample was run twice with a mobile phase of 15:9:6 (v/v/v) ethanol:butanol:water; plates were air-dried and revealed with ∝-naphthol and heat. At the same time, XOS metabolized during fermentation were evaluated using ion-exchange chromatography (HPAEC)-PAD (18). For enzymatic activity, 180 μL of non-inactivated supernatant was incubated with 180 μL of XOS and 40 μL of 0.1 M acetate buffer, pH 6.0. The reaction was incubated at 37°C and 800 rpm for 5 h. The XOS hydrolysis and xylose accumulation were confirmed by TLC and HPAEC-PAD. Standards of xylose (X1), xylobiose (X2), xylotriose (X3), xylotetraose (X4), xylopentose (X5), and xylohexose (X6) (Megazyme, Ireland) were used in all assays.

### Genome Sequencing, Assembly, and Annotation

Strains WCP-3a and WcL17 were selected for genome sequencing based on their GTF and β-xylosidase activities, respectively. DNA was extracted with the commercial UltraClean Microbial DNA Isolation Kit (MO BIO, QIAGEN) following the manufacturer’s specifications. The extracted DNA was sequenced in the Mass Sequencing Unit of the Institute of Biotechnology at Universidad Nacional Autónoma de México, in an Illumina NextSeq500 platform (2 × 75 bp) and a 550 bp insert. Adapters were eliminated with the program Trim Galore v0.4.4 (Krueger, 2015) and *de novo* assemblies were generated with Velvet v.2.2.5 (Zerbino and Birney, 2008) and SPAdes v3.11.1 (Bankevich et al., 2012); these were subsequently merged with Metassembler v1.5 (Wences and Schatz, 2015). Contigs underwent a scaffolding process with SSPACE v3.0 (Boetzer et al., 2011) and were refined with GapFiller v1.10 (Boetzer and Pirovano, 2012). Gene prediction and annotation were performed with Prokka v1.12 (Seemann, 2014).

### Taxonomic Assignment of Sequenced Genomes

Fifty-five ribosomal proteins were selected using local scripts from the sequenced genomes, as well as from representative genomes of each species of *Weissella* available in databases at the time of analysis (November 2017) (Table 1). Additionally, the housekeeping genes encoding D-Ala-D-Ala ligase (*dll*), phosphoglucomutase (*pgm*), glucose 6-phosphate dehydrogenase (*g6pd*), RNA polymerase subunit beta (*rpoB*), RNA polymerase subunit alpha (*rpoA*), and phenylalanine tRNA synthetase subunit alpha (*pheS*) were selected to construct the second phylogeny. Sequences were aligned with MUSCLE v3.8.1551 (Edgar, 2004); the prediction of the phylogenetic model was carried out with ProtTest3 (Darriba et al., 2011), and the construction of the maximum likelihood phylogeny with RaxML v8.2.10 (Stamatakis, 2014) using 100 bootstrap replicates. In addition, an average nucleotide identity (ANIm) analysis was performed with pyani v0.2.9 (Pritchard et al., 2016) using genomes representative of the genus *Weissella* and those assembled in this paper (Table 1).

**TABLE 1:** Overall properties of representative *Weissella* genomes sequenced in this work, including *W. confusa* strains WcL17 and WCP3a.

Species	Size (Mb)	Contigs	N50	Source	Origin	References (Refseq)
*W. bombi* R-53094	1.82	30	114 246	N/D	N/D	GCF_900094835.1
*W. cibaria* CH2	2.57	1	–	Fermented foods	India	GCF_001308145.2
*W. confusa* LBAE C39-2	2.28	71	63 653	Wheet sourdough	France	GCF_000239955.1
*W. confusa* DSM 20196	2.21	82	96 695	Cured sausages	China	GCF_001436895.1
*W. confusa* MBF8-1	2.18	44	84 171	Fermented soya	Indonesia	GCF_001884305.1
*W. confusa* AB3E41	2.25	54	146 189	African beer broth	Ivory coast	GCF_900166935.1
*W. confusa* WcL17	2.31	30	155 975	*Pozol*	Mex, Tab.	GCA_015594935.1
*W. confusa* WCP3a	2.22	45	119 794	*Pozol*	Mex, Chis.	GCA_015594955.1
*W. halotolerans* DSM	1.35	8	325 056	Sausages	Germany	GCF_000420365.1
*W. hellenica* R-53116	1.81	33	99 337	N/D	N/D	GCF_900095015.1
*W. jogaejeotggali* FOL01	2.14	1	–	Korean seafood	South Korea	GCF_001932615.1
*W. Kandleri* DSM 20593	1.33	36	96 380	Fermented sausages	China	GCF_001438705.1
*W. koreensis* KACC 15510	1.44	1	–	Kimchi	South Korea	GCF_000219805.1
*W. minor* DSM 20014	1.76	50	123 342	Fermented sausages	China	GCF_001437425.1
*W. oryzae* SG25	2.12	72	148 611	Fermented rice	Japan	GCF_000691805.2
*W. paramesenteroides* ATCC 33313	1.98	36	106 888	Humans	N/D	GCF_000160575.1
*W. viridescens* DSM 20410	1.53	34	86 165	N/D	N/D	GCF_001437355.1

### Comparative Genomic of Active Carbohydrate Enzymes Through the Genus *Weissella*

We performed a comparative analysis of the genomes sequenced in this paper and those representative of the genus *Weissella* (Table 1) for which the genomes discharged were re-annotated with Prokka v1.12 (Seemann, 2014). The amino acid sequences obtained were mapped with HMMER v3.1b2 hmmscan (Eddy, 2011) against the database of preserved domains of all Carbohydrate Enzymes (CAZy) families following the protocol proposed by dbCAN (Yin et al., 2012).

## Results

### Structure of the Bacterial Community of *Pozol* Over Fermentation Time

The structure and succession of bacterial communities in *pozol* were analyzed by amplicon sequencing at 0, 9, 24, and 48 h of culture. These time points represent the beginning of the fermentation process, the peak microbial growth, the shift in the microbial community and the end of that shift, respectively. From those time points, a total of 31,199 bacterial operational taxonomic units (OTUs) were obtained from all samples, distributed in 1,042 bacterial genera. As shown in Supplementary Table 1, the highest diversity in the bacterial community was found after 9 h of fermentation, and the lowest at 0 h. These results are similar to those reported in the literature on *pozol* (Ampe et al., 1999b; Ampe and Miambi, 2000; Wacher et al., 2000; Escalante et al., 2001; Bolaños et al., 2006; Cárdenas et al., 2014). According to the results on β-diversity, the 9 h and 24-h samples are similar in microbial composition (Shannon-Weaver index of 5.25 and 5.27, respectively), while the 0-h and 48-h samples are different from the rest (Shannon-Weaver index of 2.72 and 4.95) (Supplementary Table 1). The sampling procedure was successful in describing the community structure, except for the sample at 48 h (Supplementary Figure 2).

**FIGURE 1:**
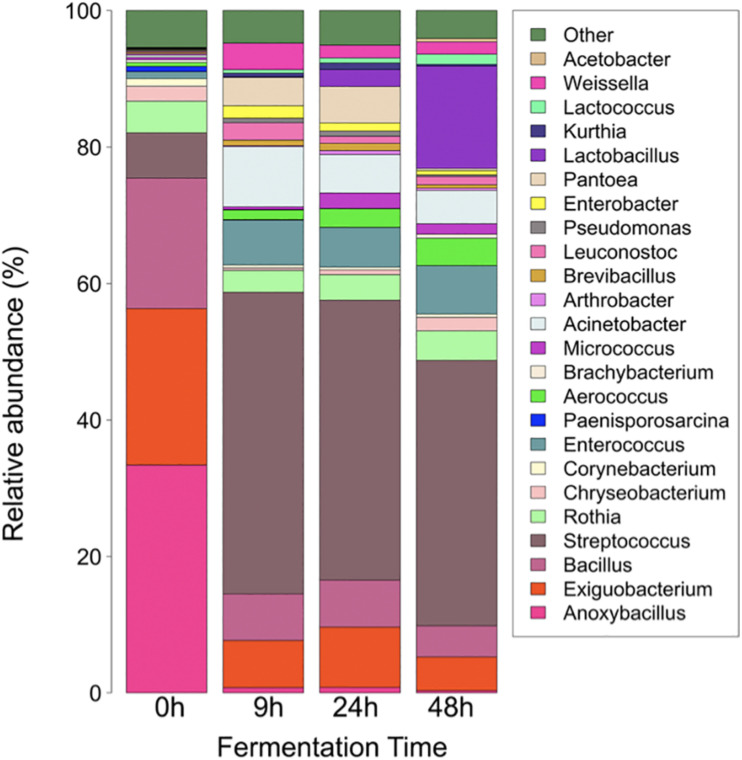
Change in the structure of *pozol* bacterial communities over fermentation time. Taxonomic assignation was made by metabarcoding (V3-V4, 16S r DNA). “Other” includes all genera with relative abundance lower than 0.05%.

As regards relative abundance, the main bacteria involved in *pozol* fermentation are lactic-acid bacteria from the phylum Firmicutes. At 0 h, the genera *Anoxybacillus, Exiguobacterium*, and *Bacillus* make more than 50% of the genera found (33.42, 22.92, and 19.11%, respectively), while *Weissella* accounted for 0.05%. On the other hand, the least represented genus was *Acetobacter*, with 0.00069%. After 9 h of fermentation, *Streptococcus* was the most abundant genus, with 44.19%, while *Weissella* ranked eighth among the 25 genera identified, with 3.89%. At 24 h of fermentation, *Streptococcus* remained as the most abundant genus, with 41.02%, while *Weissella* ranked 12th, with 1.89%. For the sample collected at the end of the process, after 48 h of fermentation, *Streptococcus* remained as the most abundant genus, with 38.94%, and *Weissella* ranked eleventh, with 1.80% (Figure 1). It is worth mentioning that *Lactobacillus* increased in relative abundance from an initial 0.07 to 15% after 48 h of *pozol* fermentation.

### Growth Kinetics and Enzymatic Activity on Birch Xylan

There was little growth of the strains studied when birch xylan was used as carbon source; the assessment of pH revealed no changes throughout the sampling period, remaining constant at 6.5 in all samples. Accordingly, xylanolytic activity was null at all sampling points (Supplementary Figure 3).

### Growth Kinetics and Xylooligosaccharide Metabolism Profile

**FIGURE 2:**
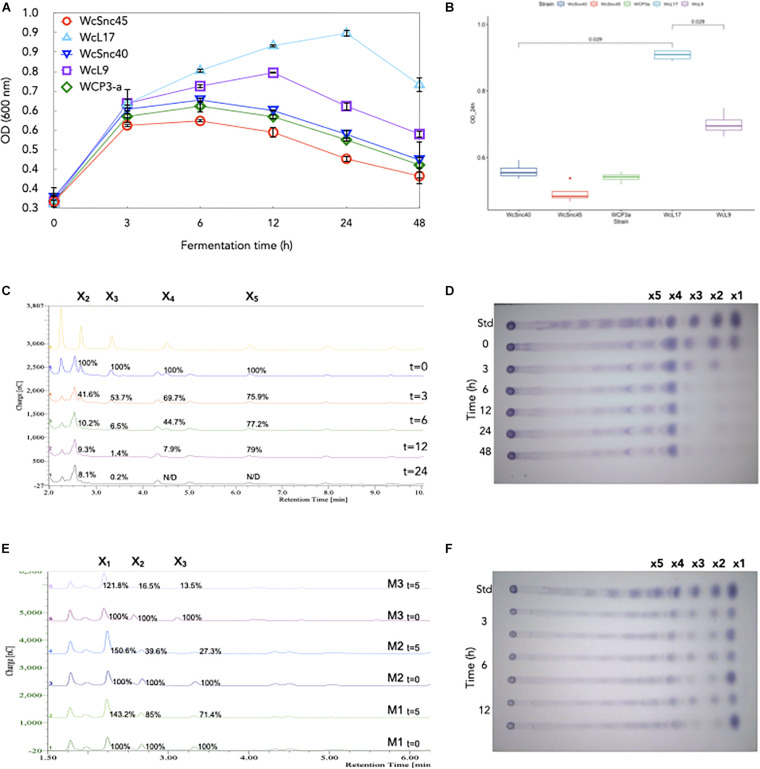
Growth and profile of xylooligosaccharides (XOS) in cultures with five *Weissella* strains isolated from *pozol*. **(A)** Biomass accumulation (OD) during fermentation. **(B)** Biomass accumulation (OD) after 24 h of growth. Statistically significant differences (*p* < 0.5) for strain WcL17 growth were obtained with the ggpubr package in R v4.0.2. XOS consumption during strain WcL17 culture was evaluated by **(C)** HPAEC-PAD chromatograms and **(D)** TLC plates. XOS hydrolysis by xylosidase activity in samples from WcL17 culture at M1 = 3 h, M2 = 6 h, and M3 = 12 h were also evaluated at baseline (*t* = 0) and after 5 h (*t* = 5) of reaction by **(E)** HPAEC-PAD chromatograms and **(F)** TLC plates.

Given the poor growth observed in birch xylan and based on the fact that the *Weissella* strains analyzed possess no amylolytic activity but can metabolize xylose (López-Hernández et al., 2018), we decided to assess their ability to metabolize XOS. As shown in Figure 2, all strains have the ability to metabolize XOS; the strain WcL17 showed the highest growth (OD_600nm_ = 0.907 ± 0.019 at 24 h), while Strain WcSnc45 showed the lowest (DO_600nm_ = 0.494 ± 0.013 after 24 h) (Figure 2A); culture pH remained constant in all cases. Hydrolysis of birch xylan produces XOS and xylose monomers, so it was important to confirm that the growth observed was due to XOS breakdown and not to xylose metabolism only. The analysis of XOS consumption followed by TLC analysis throughout the culture showed that all strains prefer to metabolize short-chain XOS, consuming almost all X_1_, X_2_, and X_3_; while X_4_ decreased more slowly over time (results not shown). Given its preferential growth (Figures 2A,B), strain WcL17 was selected to evaluate the XOS consumption kinetics by HPAEC-PAD, confirming the preferential uptake of short-chain XOS. After 24 h of growth, X_3_ and X_4_ were almost completely metabolized (91.9 and 99.8%, respectively), while X_1_ and X_2_ were consumed to a great extent (70 and 92.1%), as shown in Figures 2C,D. XOS analysis could be fully appreciated only by HPAEC-PAD. To further demonstrate the presence of the xylosidase activity in XOS WcL17 cultures, we performed reactions spiking XOS as substrate and using the 3, 6, and 12-h culture supernatants as a source of xylosidase and monitored its consumption with both TLC and HPAEC-PAD after 5 h of reaction. Again, the reduction of X_2_, X_3_, and X_4_ along with the accumulation of X_1_ (xylose) was demonstrated with both techniques (Figures 2E,F). The highest enzymatic activity was observed in 12 h-culture supernatants, showing a direct relation between xylosidase activity and growth.

### Growth Kinetics and Enzymatic Activity on Sucrose

**FIGURE 3:**
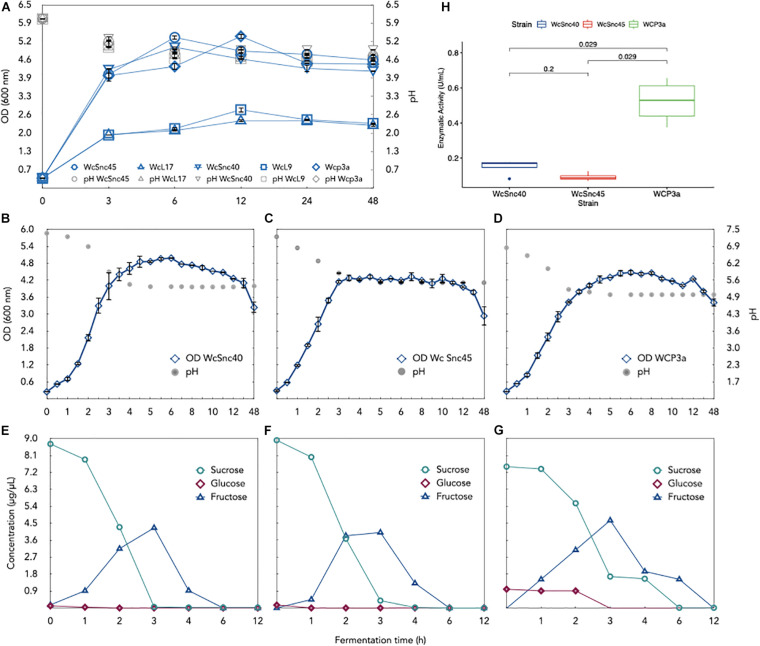
Culture of five *Weissella* strains isolated from *pozol* in MRS-sucrose medium. **(A)** Biomass evolution (OD) of the five strains. The three strains with the highest growth, namely **(B)** WcSnc40, **(C)** WcSnc45, and **(D)** WCP3a, were studied in terms of growth (OD) and evolution of pH. Metabolized sucrose, glucose, and fructose were also monitored by HPLC analysis in the three *Weissella* cultures: strains **(E)** WcSnc40, **(F)** WcSnc45, and **(G)** WCP3a. Finally, extracellular glycosyltransferase activity (U/mL) for the three strains in 6-h cultures was compared **(H)**; statistically significant differences in activity (*p* < 0.5) were obtained with the ggpubr package in R v4.0.2.

The growth profile on sucrose as substrate was evaluated in order to address another aspect related to the role of *Weissella* in *pozol* fermentation, considering that sucrose is not only found in maize seeds but also frequently added to *pozol* before consumption. It is known that *Weissella* species are able to produce polysaccharides from sucrose (20). We found that all strains assayed had the ability to grow on sucrose as carbon source; WcSnc40, WcSnc45, and WCP-3a showed greater growth and higher enzymatic activity relative to WcL17 and WcL9 (Figure 3A). A dramatic reduction in enzyme activity was also observed in supernatants after 6 h relative to 3 h of culture (Supplementary Table 2). Given this behavior, growth curves, enzymatic activity, and metabolized substrate were quantified for those strains showing high OD_600nm_. A lag phase of around 1 h was observed for WcSnc40 and WCP-3a cultures, and 0.5 h for WcSnc45. All strains ended the exponential growth and shifted to the pre-stationary phase after 3 h of culture, with peak enzymatic activity at 3 h of culture and a drop in pH after 3.5 h of culture, to remain constant thereafter (Figures 3B–D and Supplementary Table 3). Sucrose decreased during the exponential growth phase and was depleted after 6 h of culture, when strains reached the stationary phase. Interestingly, fructose release to the medium started after 1 h of culture, decreasing from 3 h onward, while glucose was readily used. This behavior suggests that glucose is used not only as carbon and energy source but also as a substrate for polysaccharide production, releasing fructose into the medium in both cases, which is metabolized once sucrose is depleted (Figures 3E–G). This was confirmed by the presence of active extracellular glucosyltransferase activity, which accounts for fructose release and, particularly, for the increase in medium viscosity, a finding not observed when XOS were used as carbon source. A fact worth mentioning is that GTF activity measured for WCP-3a on sucrose is 0.51 ± 0.13 U/mL after 3 h of culture, whereas WcSnc40 and WcSnc45 show an enzymatic activity of 0.132 ± 0.046 and 0.121 ± 0.026 U/mL, respectively, for this same time (Supplementary Table 3). Interestingly, after 4 h of culture, the enzymatic activity for the three strains falls drastically and remains stable thereafter (Supplementary Table 3). Due to its higher activity, we selected strain WCP-3a (Figure 3H) for genome sequencing.

### Genome Assembly and Taxonomic Assignment

The results obtained in the XOS and sucrose consumption kinetics experiments supported the selection of two strains for genomic sequencing, looking for genes that encode enzymatic activities on carbohydrates to explore their potential role in *pozol* fermentation. WcL17 was selected for its ability to metabolize XOS (Figure 2B), while WCP-3a was selected for its GTF activity (Figure 3H).

A total of 6,204,472 paired-end reads were obtained for WcL17 and 7,394,234 for WCP-3a. After quality- screening with TrimGalore, effective reads were reduced to 6,185,922 and 7,340,348, respectively. Thirty contigs (N50 155,975 and L50 5) and 45 contigs (N50 119,794 and L50 5) were obtained for WcL17 and WCP-3a, respectively, with an estimated genome size of 2.31 Mb (390x coverage) and 2.22 Mb (482x coverage). Gene prediction and annotation indicated a total of 2,213 coding DNA sequences (CDSs) 117 tRNAs and 6 rRNAs for WcL17; and 2,163 CDSs, 110 tRNA, and 14 rRNAs for WCP-3a. The percentage of G + C content was almost identical in both genomes, with 44.66% for WcL17 and 44.67% for WCP-3a.

**FIGURE 4:**
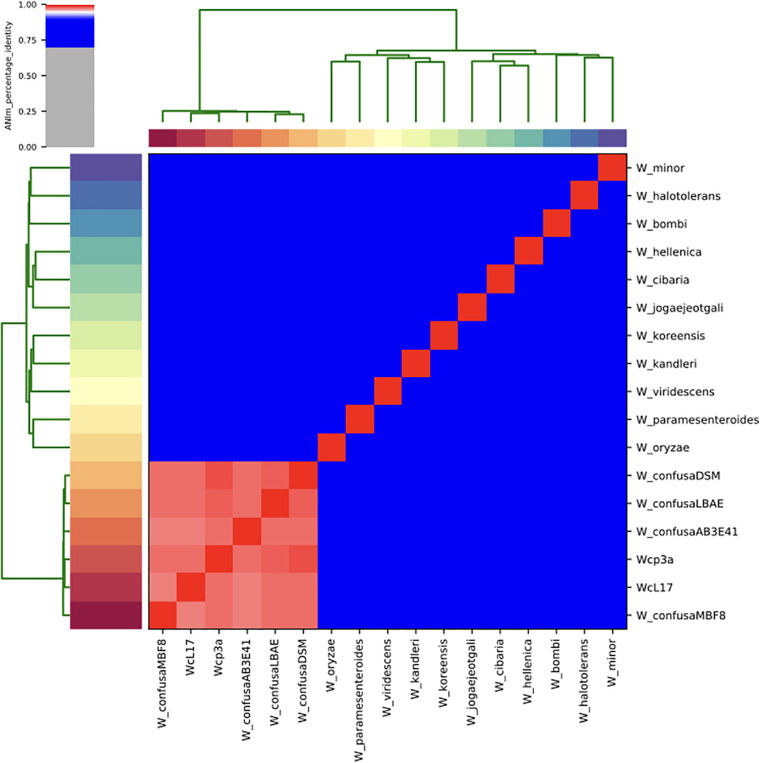
Heatmap of Average Nucleotide Identity (ANI) values of representative *Weissella* genera reported in the literature (Table 1), including genomes for strains WcL17 and WCP3a. Strains with ANI values > 90% are shown within red squares.

The taxonomic identity of the strains sequenced was confirmed through maximum likelihood analyses of housekeeping genes (Supplementary Figure 4) and 55 ribosomal proteins (Supplementary Figure 5). The support of branches clustering the strains sequenced was 100% within the *W. confusa* clade of strains from France, China, Indonesia, and Africa (Table 1). The ANIm analysis for the whole genome also confirmed that the strains WCP-3a and WcL17 correspond to *W. confusa*, with values between 97.7% and 98.5% within the species. The ANIm value between WCP-3a and WcL17 was 97.9%, whereas values between 86.9 and 87.7% were found in relation to the closest species, *W. cibaria* (Figure 4) (refer to the Github repository for the raw percentage results).

### Comparative Genomic of Active Carbohydrate Enzymes Through the Genus *Weissella*

**FIGURE 5:**
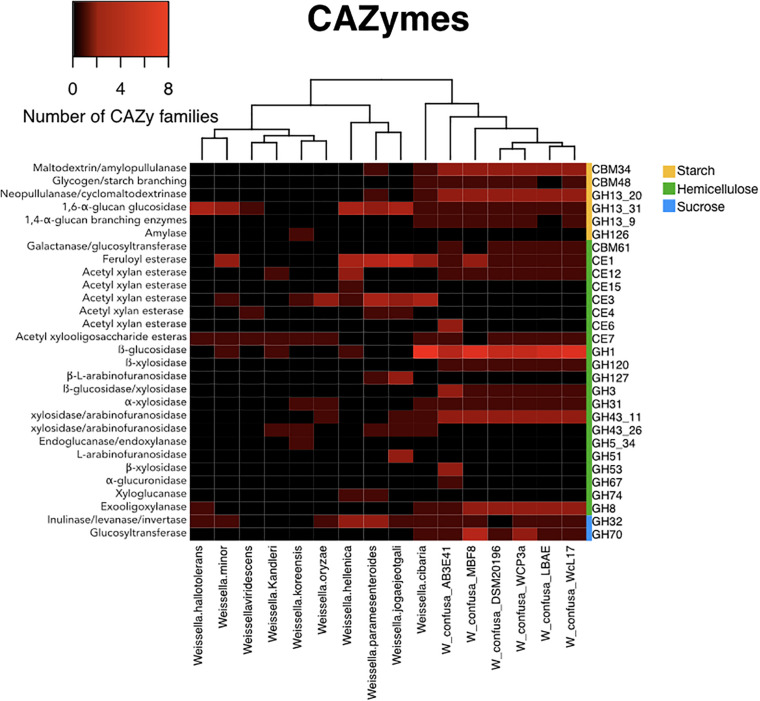
Comparative analysis of CAZy families for representative *Weissella* strains (Table 1), including WcL17 and WCP3a genomes. Colors within the map indicate the number of families in each genome, from zero to eight copies (black to red, respectively). The right sidebar indicates the type of substrate for each family (yellow = starch, green = hemicellulose, and blue = sucrose).

The search of CAZymes indicated that the genomes of WcL17 and WCP-3a encode enzymes corresponding to 43 out of the 308 families classified in the CAZy database, with a total of 87 and 79 genes from the WcL17 and WCP-3a genomes, respectively, corresponding to any of these families. These results show that *W. confusa* and *W. cibaria* contain a higher number of CAZymes analyzed relative to all species in the genus. Interestingly, this pair of species also stands out for the content of glycosyl hydrolase (GH) families acting on hemicellulose residues, together with *W. jogaejeotgali, W. paramesenteroides*, and *W. hellenica*, all isolated from fermented cereals and vegetables (Table 1). On the other hand, enzymes belonging to the GH70 family encoding GTFs were found only in *W. confusa* and *W. cibaria*, with both species already reported as producers of exopolysaccharides. Surprisingly, this analysis showed that WcL17 and WCP-3a contain genes from various subfamilies of the GH13 family, encoding a diverse group of amylases; the subfamilies present in the genomes of our strains act on starch residues. In this sense, the CAZy analysis shows a wide potential of WcL17 and WCP-3a to degrade hemicellulose and starch residues, since both strains possess genes responsible for accessory enzymatic activities, in addition to their ability to synthesize polysaccharides from sucrose (Figure 5).

## Discussion

Our results on relative abundance throughout the *pozol* fermentation process support the hypothesis that the dominant species over the process belong to the genus *Streptococcus* (Ampe et al., 1999b; ben Omar and Ampe, 2000; Wacher et al., 2000; Escalante et al., 2001; Bolaños et al., 2006; Cárdenas et al., 2014). Also, we confirmed that the genera *Enterococcus, Leuconostoc, Lactococcus*, and *Lactobacillus* are equally abundant in this process. Particularly, although not among the most numerous bacteria initially present, the abundance of *Weissella* increases steadily over the fermentation process until it attains a relative abundance similar to that reported in products such as togwa, sourdough, chicha, or fermented maize bran (Mugula et al., 2003; Bounaix et al., 2010; Elizaquível et al., 2015; Decimo et al., 2017). This may be explained by the importance of *Weissella* playing a supplementary metabolic role in the process, complementing the xylanolytic and amylolytic capacity of *S. infantarius* (Cooper-Bribiesca et al., 2018) due to the ability of *W. confusa* to break down residual oligosaccharides produced from polymers like xylan and starch during nixtamalization. This could be happening in spite of the low abundance of *Weissella*, as recently reported for the core fouling-causing microbiota in a membrane bioreactor, where low abundance taxa perform important functions (Zhang et al., 2018). This is also the case of nitrogen fixation in *pozol* carried out by *Klebsiella, Enterobacter*, and *Kosakonia* genera (Rizo et al., 2020), all of which perform important functions in the process in spite of their low abundance (<0.05%, in this work).

The limited growth and nil enzymatic activity on birch xylan became meaningful in the genomic analyses since no gene encoding endo-β-xylanase was found in the *Weissella* strains. Similar studies reported neither xylanolytic activity in strains of *W. cibaria* and *W. confusa* isolated from Indian fermented foods (idli and dahi) (Patel et al., 2013) nor growth in wheat bran xylan (Immerzeel et al., 2014). However, López-Hernández et al. (2018) reported growth of *W. confusa* L9 strain on oat xylan as the only carbon source. Our results differ from those of López Hernandez et al. probably because of the structural differences of the birch xylan used in our study, as this polysaccharide may be less susceptible to *W. confuse* enzymes, which according to their genome, could only produce an exo-oligo-xylanase (Figure 5).

Strain WcL17 showed greater growth on XOS than the rest of the strains studied, but CAZy analyses showed that WcL17 and WCP-3a have the same number and type of putative enzymes from GH families encoding XOS activities, corresponding to GH43, GH3, and GH120 for β-xylosidase and GH31 for α-xylosidase. The β-xylosidases of WcL17 are probably more efficient than those of WCP-3a; however, the characterization and subsequent comparison of these enzymes is required to address this question. On the other hand, the fact that WcL17 possesses this genetic arsenal in addition to its preference for short-chain XOS, as demonstrated by TLC and HPAEC-PAD analyses, shows that this strain has the ability to thrive in environments where this substrate is available. Such environments include *pozol*, requiring either a previous enzymatic hydrolysis of xylan by other microorganisms such as *S. infantarius* (Cooper-Bribiesca et al., 2018), or simply consuming XOS available after the alkaline treatment of maize in the nixtamalization process. The metabolism of short-chain XOS by *W. confusa* has been reported in strains isolated from fermented Indian cereals and vegetables, particularly by a β-xylosidase from the GH43 family (Patel et al., 2013; Immerzeel et al., 2014; Falck et al., 2016).

Cell growth and the presence of GTF activity demonstrate that the strains studied are able to readily degrade and process sucrose; WCP-3a displayed a higher enzymatic activity as well as a larger number of GH70 CAZymes encluding GTFs than the rest of the strains studied. This type of activity has been extensively studied in other *Weissella confusa* strains isolated from fermented foods such as sourdough wheat bran (Katina et al., 2009: Kajala et al., 2016); it is known that several strains of the *W. confusa and W. cibaria* are capable of producing dextran from sucrose, a glucose polymer also produced in fermented foods by *Lactobacillus* enzymes (Schmid et al., 2015). Among various applications, this polymer is used in the food industry because of its rheological and organoleptic properties. Strains of *Weissella confusa* have been reported to produce dextran and isomalto-oligosaccharides from the fermentation of sourdough, rye bran, and wheat bran (Katina et al., 2009: Kajala et al., 2016). We suggest that similar to other fermented foods, dextran is probably synthesized during *pozol* fermentation, conferring organoleptic and nutritional properties. Nevertheless, this aspect should be explored in future studies. Enzymes in the GH70 family are currently being characterized by our research group.

The phylogenetic (Supplementary Figures 4, 5) and ANIm (Figure 4) analyses confirmed the taxonomic identity of the genomes assembled in this investigation. The use of housekeeping markers and ribosomal proteins are an alternative to the challenges involved in taxonomic assignment based on 16S rRNA alone (Janda and Abbott, 2007) when dealing with closely related species such as *W. confusa* and *W. cibaria*, which share 87.7% of the average nucleotide identity (Björkroth et al., 2002).

The genomic analyses showed that the strains sequenced in this work have GHs acting on the substrates evaluated in the growth kinetic studies on XOS (GH43, GH3, and GH150) and sucrose (GH32 and GH70), except for xylan, in addition to genes corresponding to carboxylesterases (CE) acting on xylan decorations (CE1, CE12, and CE7). Surprisingly, the CAZy results showed alternative capacities of the WcL17 and WCP-3a strains that had not been considered previously, such as GH1 and GH8 genes encoding β-glucosidase and endo-1,4-β-glucanase, enzymes that are capable of degrading cellulose. Also interesting was the finding of the enzyme families GH13_20, GH13_31, GH13_9, which include enzymes acting on starch residues and containing the starch-binding domains CBM34 and CBM48. These findings also explain the nil detection of amylases by López-Hernández et al. (2018), as well as a potential role of starch residues in the fermentation process of the nixtamalized corn dough used in *pozol* elaboration.

Our work highlights the important supplementary metabolic role of *Weissella confusa* in the *pozol* fermentation process due to its activity on oligosaccharides derived from xylan—and eventually cellulose or starch—as well as its role in the synthesis of polysaccharides *via* GTFs.

## Data Availability Statement

Genome assemblies and raw data are available at GenBank under the BioProject PRJNA642311. Amplicon sequencing data are available under the BioProject number PRJNA648868. The code used for the all bioinformatic analysis and complete results of each are found in: https://github.com/DianaOaxaca/Weissella_analysis.git.

## Author Contributions

AL, LS, and CW designed the experiments, gave general supervision, and wrote the manuscript. DH did all the experimental work and comparative genomics. RL did sequencing 16S analysis. DH and RL wrote the initial version of the manuscript. LL supported the *Weissella* taxonomy and phylogeny study. All authors contributed to the article and approved the submitted version.

## Conflict of Interest

The authors declare that the research was conducted in the absence of any commercial or financial relationships that could be construed as a potential conflict of interest.
