# Corrigendum: Diversity of *Weissella confusa* in *Pozol* and Its Carbohydrate Metabolism

**DOI:** 10.3389/fmicb.2021.683050

**Published:** 2021-05-12

**Authors:** Diana Hernández-Oaxaca, Rafael López-Sánchez, Luis Lozano, Carmen Wacher-Rodarte, Lorenzo Segovia, Agustín López Munguía

**Affiliations:** ^1^Departamento de Ingeniería Celular y Biocatálisis, Instituto de Biotecnología, Universidad Nacional Autónoma de México (UNAM), Cuernavaca, Mexico; ^2^Programa de Genómica Evolutiva, Centro de Ciencias Genómicas, Universidad Nacional Autónoma de México (UNAM), Cuernavaca, Mexico; ^3^Departamento de Alimentos y Biotecnología, Facultad de Química, Universidad Nacional Autónoma de México (UNAM), Ciudad de México, Mexico

**Keywords:** *pozol*, maize, *Weissella*, fermented foods, CAZy

In the original article, there was an error in the Data Availability Statement as published.

The revised Data Availability Statement is below.

**Data Availability Statement**

Genome assemblies and raw data are available at GenBank under the BioProject PRJNA642311. Amplicon sequencing data are available under the BioProject number PRJNA648868. The code used for the all bioinformatic analysis and complete results of each are found in: https://github.com/DianaOaxaca/Weissella_analysis.git.

The authors apologize for this error and state that this does not change the scientific conclusions of the article in any way. The original article has been updated.

