# Supplementary material for: Diversity of Weissella confusa in Pozol and Its Carbohydrate Metabolism
Source: Front Microbiol. 2021 Mar 18;12:629449. doi: 10.3389/fmicb.2021.629449 (PMC8015861; doi:10.3389/fmicb.2021.629449)
Supplement: Supplementary file 1 [file Data_Sheet_1.PDF]

Supplementary Material

Diversity of *Weissella confusa* in *pozol* and its carbohydrates metabolism

Hernández-Oaxaca Diana[1], López-Sánchez Rafael[1], Lozano-Aguirre Beltrán Luis F.[2], Wacher-Rodarte Carmen[3], Segovia Lorenzo[1] and López Munguía Agustín[1*].

[1]Departamento de Ingeniería Celular y Biocatálisis, Instituto de Biotecnología, Universidad Nacional Autónoma de México (UNAM), Cuernavaca, Morelos, CP 62210, México.
[2]Programa de Genómica Evolutiva, Centro de Ciencias Genómicas, Universidad Nacional Autónoma de México (UNAM), Cuernavaca, Morelos, CP 62210, México.
[3]Departamento de Alimentos y Biotecnología, Facultad de Química, Universidad Nacional Autónoma de México (UNAM), Ciudad de México 04510, México.

*** Correspondence:**
Agustín López-Munguía
agustin.lopez@ibt.unam.mx

TABLE S1. Index of α-diversity of *pozol* fermentation samples

Fermentation time (h)	Observed	Chao1	se. Chao1	Shanon Weaver	Simpson
0	6 720	8 086.66	74.14	2.72	0.76
9	24 452	26 652.2	77.78	5.25	0.95
24	8 595	13 125.25	182.88	5.27	0.96
48	15 491	20 837.15	173.55	4.95	0.94

TABLE S2. Grow ($OD_{600\ nm}$), glycosyltransferase activity and pH evolution in five *Weissella* strains MRS-Sucrose cultures

	Grow ($OD_{600\ nm}$					Glycosyltransferase Activity (U/mL)					pH				
Time (h)	***Snc45***	***WcL17***	***Snc40***	***WcL9***	***WCP3a***	***Snc45***	***WcL17***	***Snc40***	***WcL9***	***WCP3a***	***Snc45***	***WcL17***	***Snc40***	***WcL9***	***WCP3a***
0	0.36 ± 0.02	0.36 ± 0.02	0.34 ± 0.02	0.35 ± 0.01	0.35 ± 0.01	N/D	N/D	N/D	N/D	N/D	6.0 ± 0.0	6.0 ± 0.0	6.0 ± 0.0	6.0 ± 0.0	6.0 ± 0.0
3	4.03 ± 0.24	1.90 ± 0.01	4.20 ± 0.02	1.88 ± 0.02	3.99 ± 0.10	0.33 ± 0.01	0.15 ± 0.01	0.25 ± 0.05	0.07 ± 0.03	0.52 ± 0.04	5.2 ± 0.0	5.0 ± 0.0	5.3±0.07	5.0 ± 0.0	5.1 ± 0.14
6	5.34 ± 0.070	2.04 ± 0.03	4.99 ± 0.01	2.11 ± 0.04	4.31 ± 0.10	0.07 ± 0.01	0.04 ± 0.01	0.12 ± 0.01	0.07 ± 0.01	0.19 ± 0.09	4.8 ± 0.0	4.7 ±0.07	4.9 ± 0.0	4.6 ± 0.0	4.9 ± 0.07
12	4.86 ± 0.06	2.38 ± 0.05	4.58 ± 0.04	2.78 ± 0.09	5.38 ± 0.08	0.08 ± 0.01	0.03 ± 0.02	0.10 ± 0.01	0.03 ± 0.01	0.11 ± 0.0	4.8 ± 0.0	4.6 ± 0.0	4.9 ± 0.0	4.6 ± 0.07	4.8 ± 0.21
24	4.74 ± 0.04	2.38 ± 0.01	4.24 ± 0.12	2.42 ± 0.02	4.42 ± 0.03	0.08 ± 0.01	0.03 ± 0.01	0.08 ± 0.01	0.03 ± 0.01	0.09 ± 0.01	4.7 ± 0.0	4.6 ± 0.0	4.9 ± 0.0	4.5 ± 0.0	4.7 ± 0.07
48	4.54 ± 0.01	2.27 ± 0.04	4.14 ± 0.02	2.29 ± 0.04	4.40 ± 0.01	N/D	N/D	N/D	N/D	N/D	4.7 ± 0.0	4.6 ± 0.0	4.9 ± 0.0	4.5 ± 0.0	4.7 ± 0.07

TABLE S3. Grow ($OD_{600\ nm}$), glycosyltransferase activity and pH evolution of three selected *Weissella* strains in MRS-Sucrose cultures.

	**Grow ($OD_{600\ nm}$)**			**pH**			**Glycosyltransferase Activity (U/mL)**		
Time (h)	***WcSnc45***	***WcSnc40***	***WCP3a***	***WcSnc45***	***WcSnc40***	***WCP3a***	***WcSnc45***	***WcSnc40***	***WCP3a***
0	0.34 ± 0.05	0.25 ± 0.02	0.27 ± 0.02	6.7	6.9 ± 0.12	6.8 ± 0.14	N/D	N/D	N/D
0.5	0.68 ± 0.04	0.52 ± 0.03	0.54 ± 0.02	N/D	N/D	N/D	N/D	N/D	N/D
1	1.39 ± 0.06	0.70 ± 0.06	0.86 ± 0.07	6.2	6.7 ± 0.10	6.5 ± 0.15	N/D	N/D	N/D
1.5	2.20± 0.06	1.24± 0.05	1.54 ± 0.11	N/D	N/D	N/D	N/D	N/D	N/D
2	3.09 ± 0.31	2.16 ± 0.13	2.19 ± 0.17	5.7	6.4 ± 0.00	6.0 ± 0.25	N/D	N/D	N/D
2.5	4.07 ± 0.11	3.29 ± 0.27	2.92 ± 0.20	N/D	N/D	N/D	N/D	N/D	N/D
3	4.83± 0.08	4.00 ± 0.49	3.42 ± 0.04	5.2	5.5 ± 0.00	5.2 ± 0.16	0.13 ± 0.05	0.12 ± 0.03	0.51 ± 0.13
3.5	4.99 ± 0.14	4.41 ± 0.24	3.79 ± 0.08	N/D	N/D	N/D	N/D	N/D	N/D
4	4.94± 0.05	4.61± 0.22	4.01 ± 0.08	4.9	5.1 ± 0.01	5.1 ± 0.06	0.08 ± 0.01	0.09 ± 0.02	0.29 ± 0.04
4.5	5.04 ± 0.09	4.86 ± 0.22	4.23 ± 0.15	N/D	N/D	N/D	N/D	N/D	N/D
5	4.89 ± 0.04	4.86 ± 0.04	4.30 ± 0.01	4.8	5.0 ± 0.02	5.0 ± 0.00	0.09 ± 0.02	0.09 ± 0.03	0.30 ± 0.05
5.5	4.96 ± 0.04	4.96 ± 0.05	4.45 ± 0.11	N/D	N/D	N/D	N/D	N/D	N/D
6	4.88 ± 0.06	4.99 ± 0.03	4.48 ± 0.07	4.8	5.0 ± 0.02	5.0 ± 0.00	0.08 ± 0.05	0.08 ± 0.02	0.29 ± 0.04
7	5.04 ± 0.18	4.77 ± 0.02	4.42 ± 0.04	4.8	5.0 ± 0.01	5.0 ± 0.01	0.08 ± 0.02	0.10 ± 0.02	0.29 ± 0.05
8	4.89 ± 0.05	4.74 ± 0.03	4.46 ± 0.05	4.8	5.0 ± 0.01	5.0 ± 0.01	0.08 ± 0.02	0.10 ± 0.04	0.28 ± 0.04
9	4.77± 0.19	4.64± 0.08	4.26 ± 0.06	4.8	5.0 ± 0.01	5.0 ± 0.01	0.08 ± 0.03	0.08 ± 0.03	0.28 ± 0.05
10	4.97 ± 0.18	4.53 ± 0.02	4.17 ± 0.03	4.8	5.0 ± 0.01	5.0 ± 0.01	0.08 ± 0.03	0.09 ± 0.02	0.29 ± 0.05
11	4.80± 0.06	4.48± 0.03	4.02 ± 0.05	4.8	5.0 ± 0.01	5.0 ± 0.01	0.08± 0.03	0.08 ± 0.03	0.29 ± 0.06
12	4.62 ± 0.05	4.27 ± 0.03	4.25 ± 0.04	4.8	5.0 ± 0.01	5.0 ± 0.01	0.08 ± 0.01	0.10 ± 0.03	0.28 ± 0.04
24	4.40 ± 0.10	4.10 ± 0.19	3.81 ± 0.05	4.8	5.0 ± 0.01	5.0 ± 0.01	0.08 ± 0.02	0.11 ± 0.031	0.27 ± 0.05
48	3.43 ± 0.41	3.24 ± 0.19	3.41 ± 0.14	4.8	5.0 ± 0.03	5.0 ± 0.01	N/D	N/D	0.23 ± 0.03

N/D= Undetermined

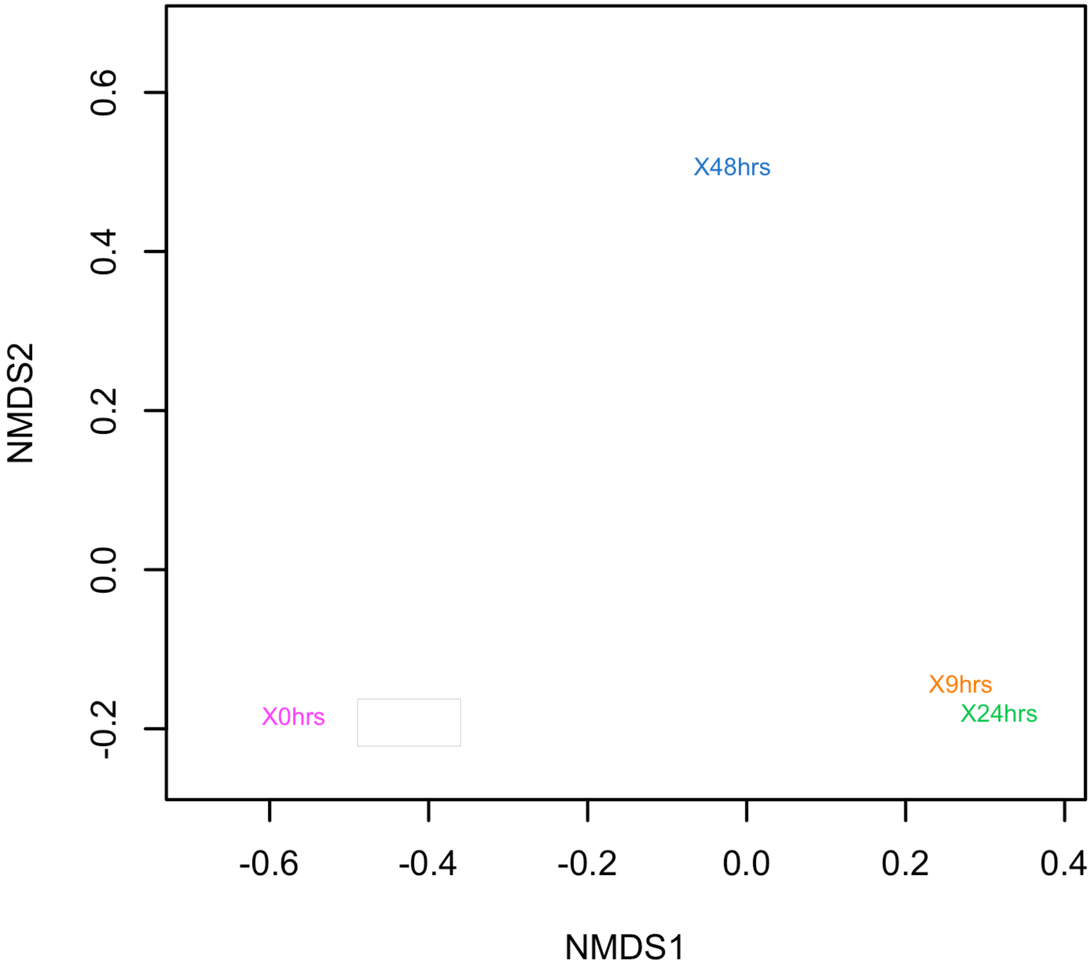

Figure S1. NMDS ß-diversity plot using the Bray-Curtis dissimilitude index. The normalized matrix was made using the R package metagenomeSeq of the samples taken at four different fermentation times: 0 (pink), 9h (orange), 24h (green), 48h (blue).

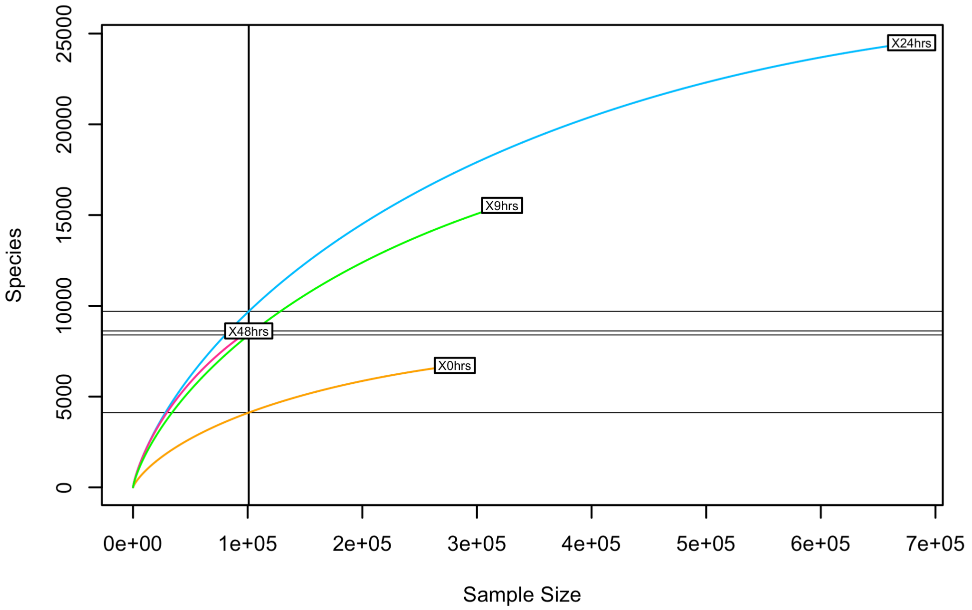

Figure S2. Sampling at four different times: 0 h (orange), 9 h (green), 24 h (blue), 48 h (red) of fermentation with 200 rarefactions. Figure made with the R vegan Package.

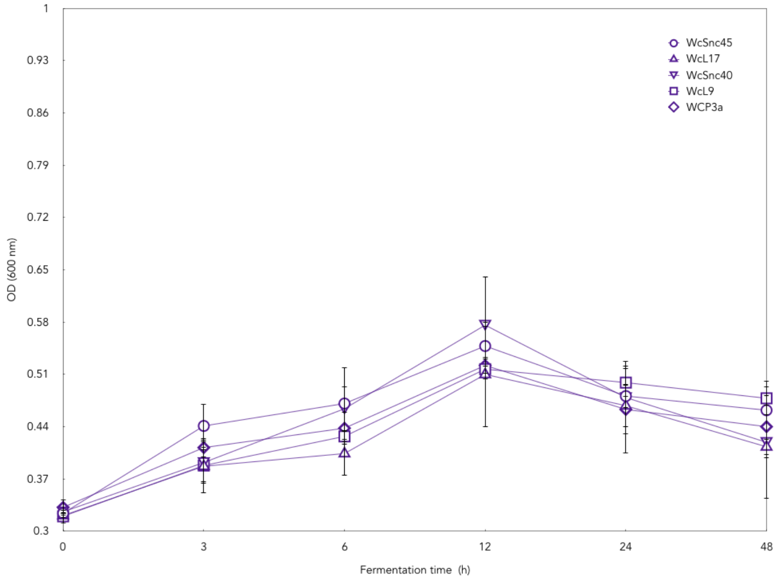

Figure S3. Growth evolution in MRS-Xylan medium of five *Weissella* strains isolated from *pozol*. Vertical lines correspond to the standard deviation of samples. Details of culture conditions are described in the Material and Methods.

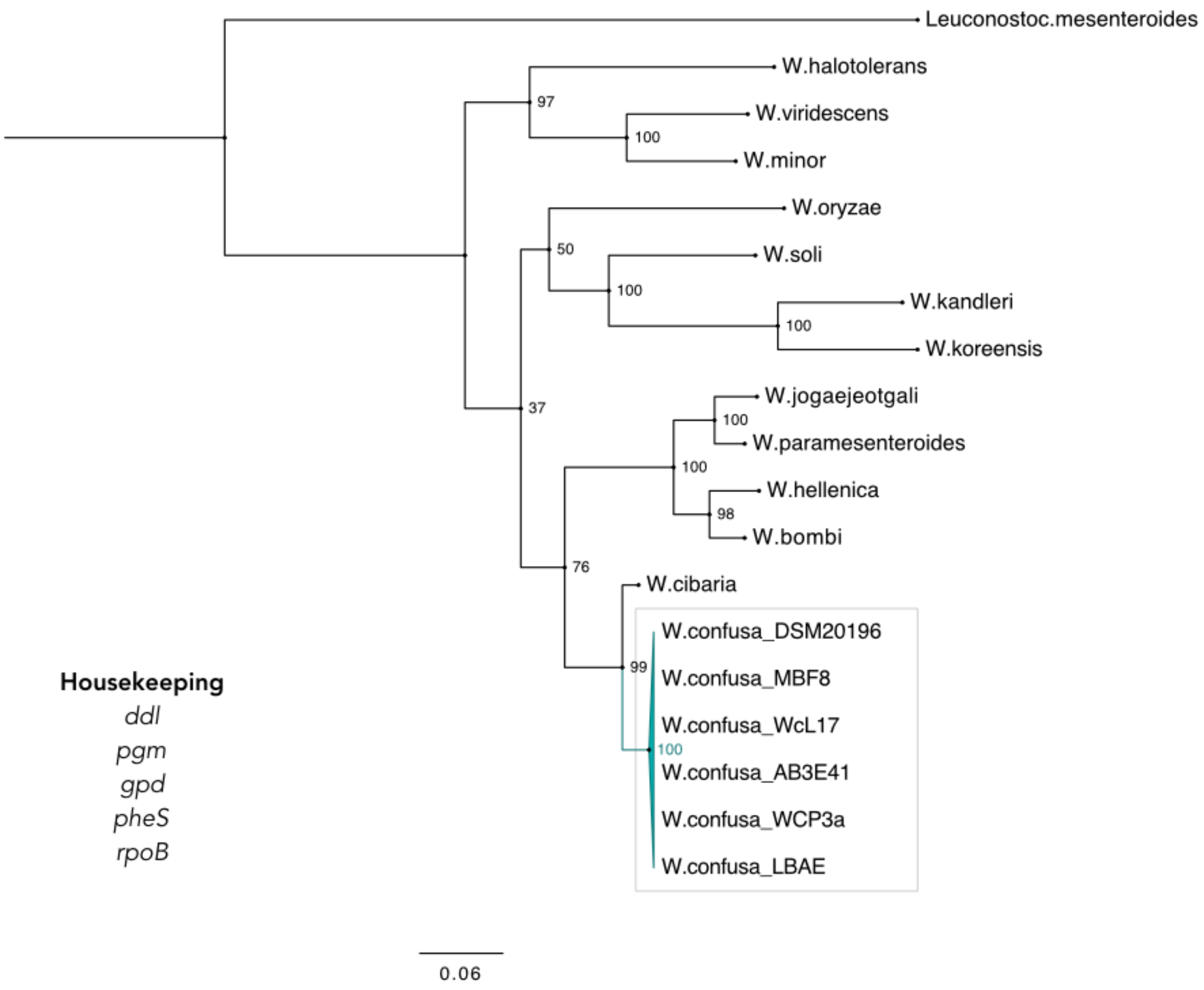

Figure S4. Phylogenetic analysis of housekeeping genes of genus *Weissella* (Table 1). The blue color indicates that WCP-3a and WcL17 are grouped in to the *Weissella confusa* clade.

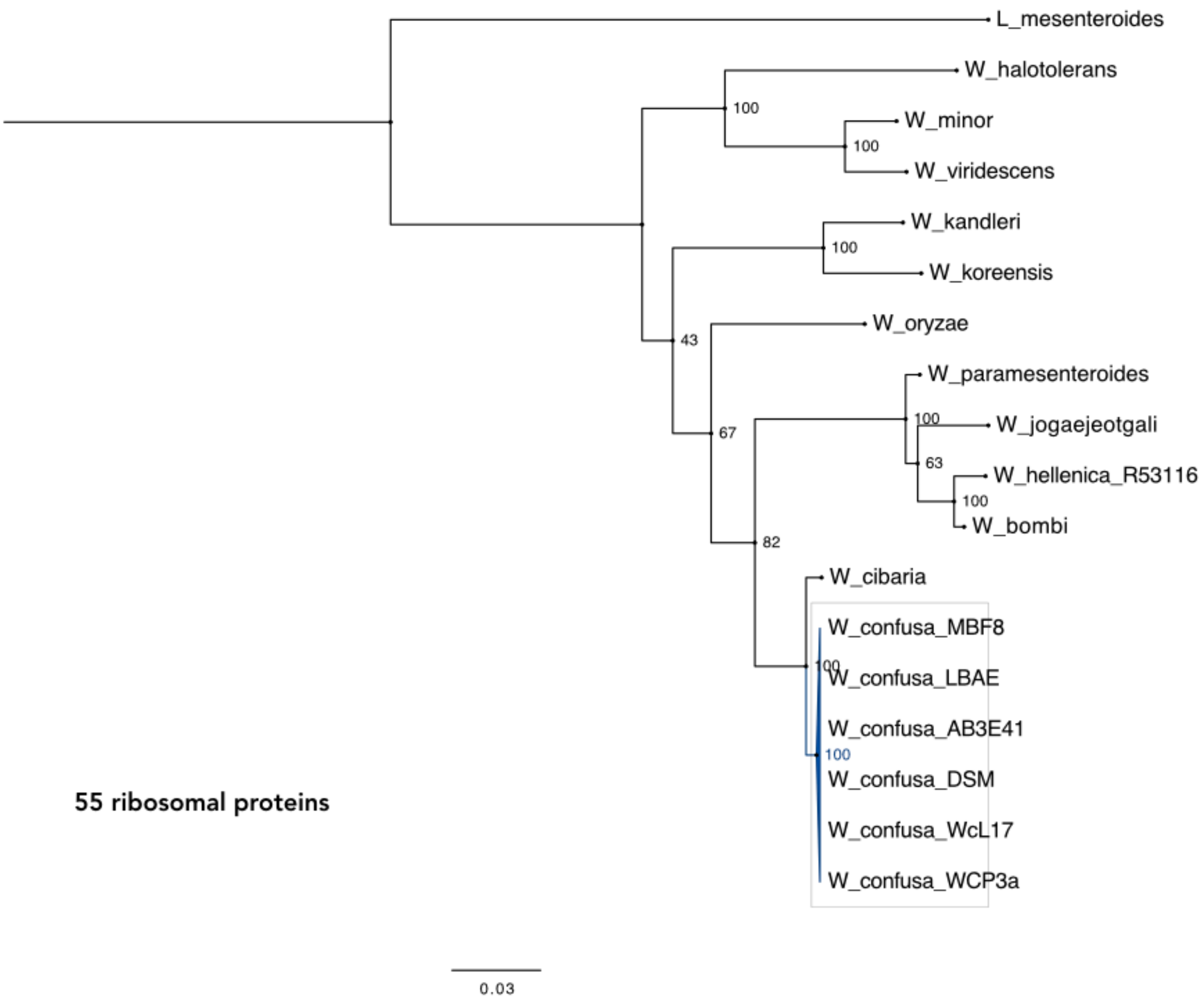

Figure S5. Phylogenetic analysis of fifty-five ribosomal proteins of genus *Weissella* (Table 1). The blue color indicates that WCP-3a and WcL17 are grouped in to the *Weissella confusa* clade.
